# The Effectiveness of NP001 on the Long-Term Survival of Patients with Amyotrophic Lateral Sclerosis

**DOI:** 10.3390/biomedicines12102367

**Published:** 2024-10-16

**Authors:** Bruce D. Forrest, Namita A. Goyal, Thomas R. Fleming, Paige M. Bracci, Neil R. Brett, Zaeem Khan, Michelle Robinson, Ari Azhir, Michael McGrath

**Affiliations:** 1 Hudson Innovations, LLC, Nyack, NY 10960, USA; 2 Neuvivo, Inc., Palo Alto, CA 94301, USAmike@neuvivo.com (M.M.); 3 School of Medicine, University of California, Irvine, CA 92697, USA; 4 School of Public Health, University of Washington, Seattle, WA 98195, USA; 5 Department of Epidemiology and Biostatistics, University of California, San Francisco, CA 94143, USA; 6 Pharmaceutical Product Development, LLC, Montreal, QC H4T 1V6, Canada; 7 Engage Management Solutions, LLC, Hillsborough, NC 27278, USA

**Keywords:** amyotrophic lateral sclerosis, innate immunity, neuroinflammation, sodium chlorite, overall survival

## Abstract

Background/Objectives: The aim of this study was to estimate the effect of a 6 months’ treatment course of the innate immune modulator NP001 (a pH-adjusted intravenous formulation of purified sodium chlorite), on disease progression, as measured by overall survival (OS) in patients with amyotrophic lateral sclerosis. Methods: Blinded survival data were retrospectively collected for 268 of the 273 patients who had participated in two phase 2 placebo-controlled clinical trials of NP001 (ClinicalTrials.gov: NCT01281631 and NCT02794857) and received at least one dose of either 1 mg/kg or 2 mg/kg of NP001 as chlorite based on actual body weight, or placebo. Kaplan–Meier methods were used on the intent-to-treat population to estimate survival probabilities. Results: In the overall population, the median OS was 4.8 months (2.7 years [95% CI: 2.3, 3.5] in the 2 mg/kg NP001group and 2.3 years [95% CI: 1.8, 2.9] in the placebo group). Hazard ratio (HR): 0.77 (95% CI: 0.57, 1.03), *p* = 0.073. Among patients aged ≤ 65 years, the median OS for the 2 mg/kg NP001 group was 10.8 months (3.3 years [95% CI: 2.4, 3.8] in the 2 mg/kg NP001 group and 2.4 years [95% CI: 1.7, 3.3] in the placebo group). HR: 0.69 (95% CI: 0.50, 0.95). No differences were observed in the 1 mg/kg NP001 group or in patients aged > 65 years. Conclusions: The findings from this study suggest that a 6 months’ treatment course of NP001 resulted in a 4.8-month increase in overall survival in patients with ALS. The findings from this study indicate that targeting inflammation associated with the innate immune system may provide a pathway for new therapeutic options for the treatment of ALS.

## 1. Introduction

Amyotrophic lateral sclerosis (ALS), also known as motor neurone disease (MND), is a relentlessly progressive and fatal chronic inflammatory neurodegenerative disease primarily characterized by the progressive deterioration of cortical and spinal motor neurons [1,2]. The loss of motor neurons manifests as muscle weakness leading to death of most patients within 20–48 months after diagnosis [3], with the median survival time for patients aged less than 65 years reported to be 40.2 months compared with 25.9 months for patients aged ≥ 65 years [4].

The mechanism of action of NP001 as a modulator of inflammation in ALS driven by the innate immune system is described by McGrath et al. [2]. Briefly, NP001 is a pH-adjusted intravenous formulation of purified sodium chlorite, a first-in-class molecule with a novel mechanism of action that regulates inflammation [2,5].

In two previous phase 2 studies of NP001 [6,7], the primary outcome measures of change in the slope of the amyotrophic lateral sclerosis functional rating scale-revised (ALSFRS-R) total score over time suggested modest effects for NP001 that did not achieve statistical significance. Data from these initial analyses suggested that the effects on ALSFRS-R and other available measures might be more favorable in patients ≤ 65 years of age. Given their sample sizes, both studies were statistically underpowered for effects on ALSFRS-R and certainly for effects on overall survival (OS), and thus would not reliably distinguish ineffective from moderately effective interventions [8].

An adequately powered study for an endpoint such as OS would require a sufficient number of OS events, achievable by recruiting more patients, especially patients at higher risk, and/or by obtaining longer-term follow-up. Obtaining longer-term follow-up for an endpoint such as OS could be meaningfully more informative, since initial data analyses for both trials had OS follow-ups only at approximately 6 months. The intent of this study was to estimate the effect of NP001 on OS, with this assessment based on OS data that had not been previously collected nor analyzed [2].

## 2. Materials and Methods

### 2.1. Study Patients

This study was designed to collect survival data for all 273 patients who had previously been randomized in two phase 2 clinical studies received at least one dose of one of two dose levels of NP001 or placebo. NP001 is a pH-stabilized intravenously administered formulation of sodium chlorite. Full details of these two previous clinical studies have been reported previously [6,7]. The phase 2 clinical studies were as follows: (a) phase 2a (NCT01281631), performed between 24 March 2011 and 25 September 2012, in which a total of 136 patients were enrolled and randomized to receive either 1 mg/kg of chlorite (n = 49), 2 mg/kg of chlorite (n = 45), or normal saline as a placebo (n = 42); and (b) phase 2b (NCT02794857), performed between 29 August 2016 and 12 December 2017, in which a total of 138 patients were enrolled and randomized to receive either 2 mg/kg NP001 (n = 70) or normal saline as the placebo (n = 68). The dose of NP001 administered was calculated as the dose of chlorite based on actual body weight; all doses will be referred to as the dose of chlorite administered.

In both studies, all patients were diagnosed with ALS using the El Escorial criteria categories possibly, probably, or definite and were enrolled within 3 years of symptom onset. Additionally, vital capacity had to be >70% forced vital capacity (FVC) for the phase 2a study and >65% slow vital capacity (SVC) for the phase 2b study. FVC and SVC are highly correlated and both FVC and SVC measurements and their loss over time serve as important biomarkers related to the quality of life and mortality in ALS patients [9]. Each patient was scheduled to receive six (6) dosing cycles of the applicable study drug over a 6-months’ treatment period [6,7].

In addition, all patients enrolled in the phase 2b study were required to have a plasma high-sensitivity C-reactive protein (hs-CRP) concentration of ≥0.113 mg/dL at the pre-screening visit. Elevated CRP levels are commonly used as a marker of underlying inflammation [10].

Key demographic data (i.e., age, sex, race, and ethnicity) were taken from the data already collected for those original clinical studies.

### 2.2. Study Design and Data Collection

This was a retrospective study involving the original 25 phase 2 study sites: 24 in the USA and 1 in Canada. Sites were asked to provide information about existing patient survival status, date of death, and cause of death, if known, coded with the same unique, de-identified patient number assigned in the respective phase 2 study. A review of survival status data from medical records of the treating institutions’ patient charts was performed to identify dates of death or last known date alive, which included a review of the primary record to determine that adequate source documentation was available to support reported data; and review of data collection forms to determine accurate reporting of research data. Where such data were not available, survival status data were identified using publicly available records and databases, such as the Social Security Death Index, State death indexes, and obituary resources; these were also used to identify patient dates of deaths. For the purposes of this study, the cut-off for the determination of the outcomes was 30 September 2022, (longest follow-up: 11 years and 8 months after randomization), in order to incorporate the most mature data to date to enhance currentness of the analysis of OS.

If OS data were captured through 30 September 2022, for 95% of the 110 patients receiving placebo and 115 patients 2 mg/kg of chlorite (i.e., at least 214 of these 225 patients), assuming a 48-month median OS in the pooled dose groups, this update would provide 150 death events overall and 110 deaths in the subgroup aged ≤ 65 years.

- With 150 OS endpoints, statistical significance would be obtained with an estimated 2 mg/kg of chlorite vs. placebo OS HR ≤ 0.726. This would be achieved with 80% power if the true OS HR = 0.633.
- With 110 OS endpoints, statistical significance would be obtained with an estimated 2 mg/kg of chlorite vs. placebo OS HR ≤ 0.688. This would be achieved with 80% power if the true OS HR = 0.586.

Such a sample size affords adequate statistical power to address the study objective.

Data collection was performed by Omnitrace Corp. (Palm Beach Gardens, FL, USA), a firm specializing in locating participants and ascertaining the vital status through searches of public records, obituary databases, property records, and social media, which was blinded to treatment assignment during data collection activities. The study was managed by Pharmaceutical Product Development, LLC (PPD), part of Thermo Fisher Scientific, Inc., (Wilmington, NC, USA), which also remain blinded to treatment assignment during data cleaning and analysis activities.

Data collection began on 7 December 2022, and database lock occurred on 11 December 2023.

### 2.3. Study Endpoints

The primary endpoint analysis was the determination of OS in all patients who received at least one infusion of NP001 at a dose of 2 mg/kg of chlorite, compared with the placebo. Supportive analyses included assessment of effects on OS in subgroups by age ≤ 65 years vs. >65 years who received at least one infusion of NP001 at a dose of 2 mg/kg of chlorite compared with the placebo; and effects on OS in all patients who received at least one infusion of NP001 at a dose of 1 mg/kg of chlorite compared with concurrently randomized placebo patients.

These analyses for effects on OS were responsive to the analyses of available data from the Phase 2a and Phase 2b trials that revealed the effects of NP001 on the primary and secondary endpoints of these trials tended to be more favorable on the 2 mg/kg arm than the 1 mg/kg arm, and in 2 mg/kg arm patients who were ≤65 years old.

### 2.4. Statistical Analysis

The overall analysis population (i.e., ITT population) was defined as all patients with ALS who were enrolled in this study and had previously received a dose of NP001 or placebo.

The key analysis outcome was overall survival, which was defined as the time from the original random assignment to the date of death due to any cause (event). Patients without documented deaths prior to the end of the study follow-up period (i.e., 30 September 2022) were censored at their last contact date or 30 September 2022. Overall survival was censored for patients at the date of randomization if they were randomized but had no follow-up.

Patient demographic/characteristic data from the clinical studies were merged with the collected survival data at the time of analyses. The original study patient ID number and group assignment were collected to merge the datasets appropriately. In the case of discrepant data between the current study CRF data and original clinical study data, the original clinical study data were considered the primary data source.

Because the data from the original phase 2 clinical studies had been unblinded, before data from the current study were shared with the study team, variables (i.e., group assignment) were blinded by a data analyst outside of the study team. All study-level analyses were performed blinded, and the study team was only unblinded after the final study-level results were produced. To make appropriate group comparisons across studies, the data analyst on the study team was unblinded after the study-level results were finalized and then proceeded with the integrated analysis across the two clinical studies.

For descriptive patient characteristics, continuous data were described by mean, standard deviation (SD), median, interquartile range (including first and third quartiles), minimum, maximum, number of known and number of unknown (missing) observations. Categorical variables were described by frequency and percentages (n, %). Percentages were calculated using the specified denominator in the table.

Kaplan–Meier methods were used to estimate survival probabilities and curves. For the comparison of the 2 mg/kg of chlorite vs. placebo groups, the stratified log-rank statistic was used as the primary analysis to assess differences in overall survival between the treatment and placebo groups for the ITT population, where stratification was by the original study (i.e., phase 2a vs. phase 2b). For the comparison of the 1 mg/kg of chlorite vs. placebo groups, since data were only from the phase 2a study, the log-rank statistic without stratification by study was used. Supportive analyses for the comparison of 2 mg/kg of chlorite vs. placebo groups included separate analyses by study using the log-rank statistic.

For each outcome analysis, the number and percentage of patients with the event, the number and percentage of patients censored, and Kaplan–Meier estimates of the quartiles of the survival distribution along with corresponding 95% confidence intervals were calculated. Regarding additional descriptive analyses, the hazard ratio (HR) and corresponding 95% confidence interval comparing NP001 to placebo were estimated using a Cox proportional hazards model, stratified by the original study (phase 2a, phase 2b). Descriptive assessments were performed regarding the assumption of proportionality of hazard ratios, and influential observations were assessed. All *p*-values were two-sided. The analyses using the stratified Cox proportional hazards model were based on the Wald statistic from the Cox partial likelihood and were only descriptive, in contrast to the previously specified primary analyses based on the stratified log-rank statistic, that are based on the score statistic from the Cox partial likelihood. All time-to-event analyses were reported in years. The random assignment date was ascertained from the original study data.

## 3. Results

Of the combined 274 patients originally randomized in the Phase 2 studies, 273 received either NP001 or the placebo, and the date of death or last known date alive could be discerned for 268 (98.2%) of those patients. One patient had been randomized but did not receive a dose of either NP001 or the placebo. With respect to the other five patients for whom no data could be found, the sponsor had randomization codes for these patients; however, the sites were unable to match available patient identifiers with any corresponding patient medical record in their institution’s electronic records database.

### 3.1. Baseline Characteristics of Patients

Baseline characteristics for the NP001 and placebo recipients for each of the two original studies are presented in Table 1. Due to the time at which the original studies were performed, no information on genetic mutations that may be associated with ALS disease progression was available.

Across both studies, the overall median age at time of randomization was 57 years old, and the interquartile range spanned from 48 to 63 years old. The majority of patients were male (68.9%), non-Hispanic or Latino (94.5%), and White (94.1%). Most patients (90.8%) had sporadic ALS with site of ALS onset in the limbs (85.0%). Overall, the median duration of ALS symptom onset was 18.3 months. The majority of patients across both studies had prior riluzole use (75.5%) and were concurrent riluzole users (69.2%) at the time of their enrollment in the original studies.

### 3.2. Sources of Patient Survival Status Data

The primary source of survival status in this study was the patient’s medical record (79.9%) followed by the Social Security Death Index (11.9%), obituary resources (4.5%), other sources such as a state death index, a property sale, grave records (1.9%), motor vehicle registration (1.1%), Driver’s License Registration (0.4%) and Property Tax records (0.4%).

### 3.3. Overall Survival in Patients Receiving 2 Mg/Kg NP001 vs. Placebo

**Figure 1:**
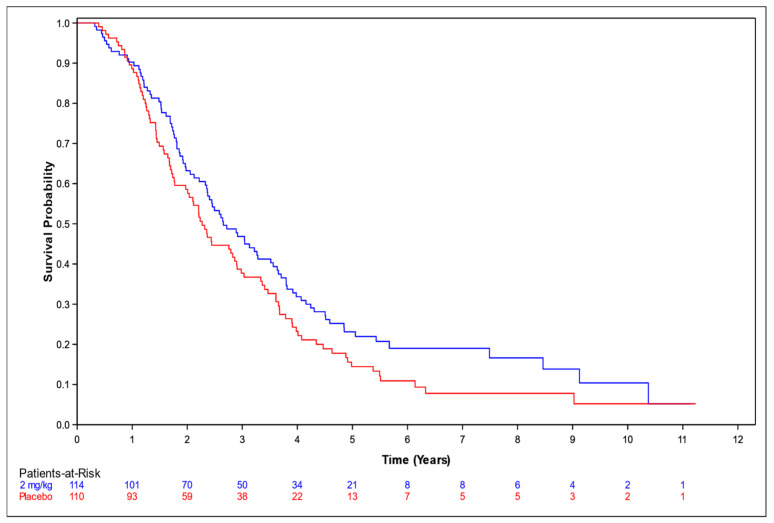
Kaplan–Meier curve of survival probability for patients who received NP001 at a dose of 2 mg/kg of chlorite compared with placebo. Blue line: 2 mg/kg dose, median survival 2.7 years (95% CI: 2.3, 3.5); red line: placebo, median survival 2.3 years (95% CI: 1.8, 2.9).

**Figure 2:**
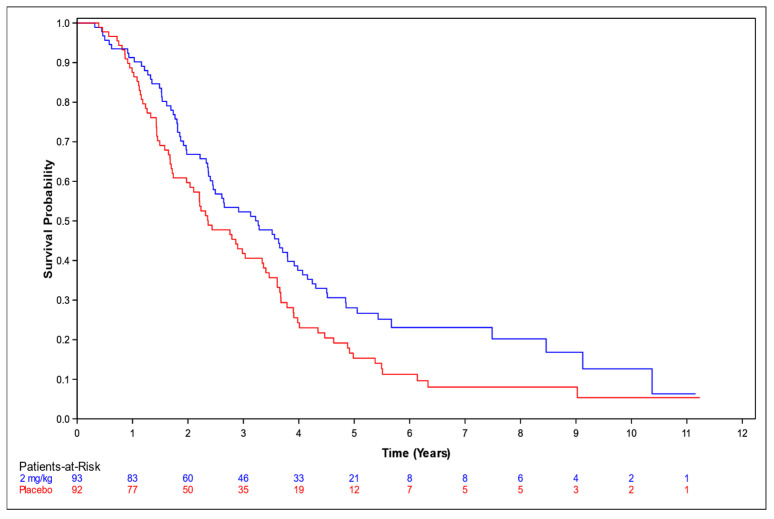
Kaplan–Meier curve of survival probability for patients ≤65 years old who received NP001 at a dose of 2 mg/kg compared with placebo. Blue line: 2 mg/kg dose, median survival 3.3 years (95% CI: 2.4, 3.9); red line: placebo, median survival 2.4 years (95% CI: 1.7, 3.3).

**Figure 3:**
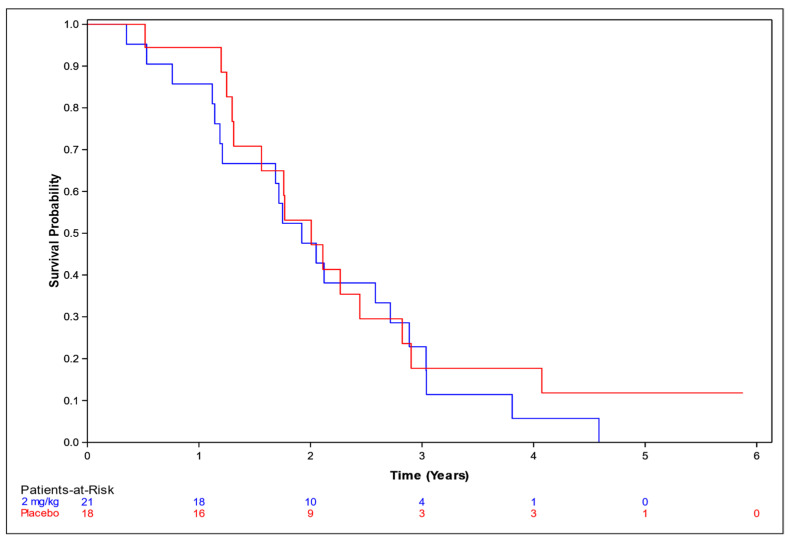
Kaplan–Meier curve of survival probability for patients >65 years old who received NP001 at a dose of 2 mg/kg compared with placebo. Blue line: 2 mg/kg dose, median survival 2.2 years (95% CI: 1.7, 3.4); red line: placebo, median survival 2.1 years (95% CI: 1.6, 3.4).

OS among patients who received NP001 at a dose of 2 mg/kg chlorite compared to placebo was assessed with respect to the overall population (Figure 1), and in supportive analyses in the ≤65 years and >65 years subsets (Figure 2 and Figure 3, respectively).

In the overall population, the median survival (95% confidence interval [CI]) over the entire follow-up duration was 2.7 years (95% CI: 2.3, 3.5) and 2.3 years (95% CI: 1.8, 2.9) in the 2 mg/kg chlorite and placebo groups, respectively (*p* = 0.073). The associated HR was 0.77 (95% CI: 0.57, 1.03).

Among those aged ≤ 65 years, the median OS was 3.3 years (95% CI: 2.4, 3.9) in the 2 mg/kg group and 2.4 years (95% CI: 1.7, 3.3) in the placebo group, representing a 10.8-months’ advantage for the NP001 recipients. The associated HR was 0.69 (95% CI: 0.50, 0.95). Among those aged > 65 years, the median OS was 1.9 years (95% CI: 1.2, 2.7) in the 2 mg/kg group and 2.0 years (95% CI: 1.3, 2.8) in the placebo group. The associated HR was 1.02 (95% CI: 0.54, 2.16).

### 3.4. Overall Survival in Patients Receiving 1 Mg/Kg NP001 vs. Placebo

OS among patients who received 1 mg/kg of chlorite compared to the placebo was also assessed among the overall population. The median survival over the entire follow-up duration was 2.2 years (95% CI: 1.7, 3.4) in the 1 mg/kg group and 2.1 years (95% CI: 1.6, 3.4) in the placebo group. The associated HR was 0.92 (95% CI: 0.59, 1.43).

## 4. Discussion

In this analysis of survival encompassing patients randomized in the two original phase 2 studies of NP001, those patients randomized to receive a 6-months’ treatment course of 2 mg/kg of chlorite had a median OS for the primary analysis that was 4.8 months longer than the group randomized to receive the placebo. In supportive analyses, those patients aged ≤ 65 years and randomized to receive a 6-months’ treatment course of 2 mg/kg of chlorite had a median OS that was 10.8 months longer than the group randomized to receive the placebo; in contrast, in patients aged greater than 65 years and randomized to receive a 6-months’ treatment course of 2 mg/kg of chlorite, there was no difference in OS compared with those randomized to receive the placebo. The absence of an observable benefit in those patients receiving the lower dose of 1 mg/kg of chlorite suggests a dose–response relationship for efficacy. When considered in the broader context of reported analyses of the effect of NP001 on vital capacity, ALSFRS-R total score and inflammatory biomarkers [2,5], these analyses of newly collected survival data provide supportive evidence of the potential treatment effect of NP001 in ALS in extending life.

The ALSFRS-R is a rating instrument for monitoring the progression of disability in patients with amyotrophic lateral sclerosis (ALS) and has been widely used as an outcome measure in ALS clinical practice and clinical studies as a primary or secondary outcome measure, acting as an alternative to OS, on the basis that the total score may provide the opportunity to identify an earlier outcome, thus shortening the duration of clinical studies in patients with ALS [11].

However, the generalizability of changes in the total ALSFRS-R score is unclear; there is a lack of clarity with respect to what is to be considered a clinically meaningful change in the slope of the total ALSFRS-R score in response to treatment; issues of non-linearity, multidimensionality and floor and ceiling effects have challenged the continued utility of the ALSFRS-R as a primary outcome measure [12,13,14].

Because of its unequivocal clinical relevance, survival was used as the primary outcome measure in evaluating new potential therapies for ALS in early clinical studies [15,16,17,18], and it provides a definitive outcome not afforded by the ALSFRS-R total score. While composite endpoints such as tracheostomy, permanent assisted ventilation or death allow more study participants to reach endpoint events for data analysis, especially for patients aged 65 years or older [19], this approach introduces unintended factors in the analysis such as variation in clinician treatment practices or participant acceptance of ventilatory interventions [20]. Gordon et al. determined that tracheostomy and permanent assisted ventilation were not equivalent to death in ALS because respiratory interventions differed between centers, leading to variability in combined outcome assessments, such that the time to the endpoint could differ significantly depending on its definition. They concluded that the death rate alone is the least variable and most easily identifiable measure of survival rate in ALS [20].

At the time of the patients’ participation in the original two phase 2 clinical studies, only riluzole was approved for the treatment of ALS, and the patient exposure to riluzole was balanced at baseline between the NP001 and placebo groups in both studies. Riluzole is regarded as influencing the time to patient death, based on two randomized, double-blind, placebo-controlled clinical studies where the clinical outcome measure was time to tracheostomy or death over a follow-up period of at least 12 or 13 months, respectively (up to a maximum duration of 18 months). The reported increase in survival for patients with stage 4 disease was 2–3 months, based on the analysis specified in the study protocols (Logrank test *p* = 0.12 and *p* = 0.076, respectively) [21].

However, it is possible that in the period following completion of the active treatment phase of the phase 2 studies, some longer-term survivors may have been exposed to edaravone, which was approved in 2019. Despite edaravone having some indication of a treatment benefit in slowing the rate of decline in the ALSFRS-R total score in a subset of approximately 8% of all ALS patients [22], it has not been shown to have any effect on patient survival nor disease progression in a post-approval phase 3 study [23]. It is unlikely that any of the surviving participants in the NP001 phase 2 studies had any exposure to the fixed dose combination of sodium phenylbutyrate and taurursodiol, which, despite some indication of an effect on patient disease progression as determined in a post hoc analysis of a single phase 2 study and an open label extension [24], has recently been reported to have no treatment effect in a phase 3 study [25].

The current survival data collected in this study were analyzed using an ITT methodology. The application of an ITT analysis provides an unbiased estimate of treatment effect because it includes all patients in the groups to which they were randomly assigned, regardless of post-randomization intercurrent events or deviations from the protocol [26,27,28], and avoids biased overestimates of the efficacy of an intervention resulting from the removal of non-compliers by accepting that noncompliance and protocol deviations are likely to occur in actual clinical practice [29]. In this instance, the application of the ITT analysis accepts the real-world nature of clinical practice and the anticipated treatment effect of NP001 irrespective of alternative treatments, therapeutic agents, or permanent assisted ventilation.

With respect to the limitations of this study, the survival status for patients who were previously enrolled in two phase 2 clinical studies was determined and confirmed through the review of patient medical records, and in cases where data were not available, through research of publicly available records and databases. The studies were completed on 25 September 2012 and 12 December 2017, respectively. The length of time that elapsed since the closure of these studies contributed to the challenges in data collection, which included the departures of the original investigator and/or site staff from the sites, and the limited number of site staff to retrieve and provide the required data to Omnitrace. However, given that the data collection form required a minimum amount of data to be collected, the impact on data quality was assumed to be minimal. Furthermore, OS is well established as an objectively measured, unambiguous, clinically significant outcome—unaffected by the timing of assessment and not subject to the potential investigator biases associated with outcomes that require clinical judgment. This further minimized the limitations associated with data collection. It should also be noted that, in both phase 2 studies, the majority of patients in both the treatment and placebo cohorts received concurrent riluzole, and survival outcomes in the current study did not adjust for the potential effect of riluzole on survival probabilities.

## 5. Conclusions

The findings reported here suggest that just a 6-months’ treatment course of NP001 has an important effect on overall survival, an objectively measured, unambiguous, clinically significant outcome unaffected by assessment timing and not prone to the potential investigator biases associated with endpoints that require clinical judgment. Further, this study indicates that targeting inflammation associated with the innate immune system may provide a pathway for new therapeutic options for the treatment of ALS.

## Figures and Tables

**Table 1:** Baseline Demographic Characteristics for Patients Who Received at Least One Infusion of NP001.

	Overall	Phase 2a			Phase 2b	
	(n = 273)	2 mg/kg (n = 45)	1 mg/kg (n = 49)	Placebo (n = 42)	2 mg/kg (n = 69)	Placebo (n = 68)
**Characteristics**						
**Patients included from the original trials, n (%)**	268 (98.2%)	44 (97.8%)	47 (95.9%)	40 (95.2%)	69 (100.0%)	68 (100.0%)
*Patients not included, n (%)*	5 (1.8%)	1 (2.2%)	2 (4.1%)	2 (4.8%)	0 (0.0%)	0 (0.0%)
**Age (years)**						
Mean (SD)	56 (10.9)	54 (10.2)	54 (12.4)	54 (9.5)	58 (10.9)	57 (10.7)
Median	57	54	56	55	59	57
(Q1:Q3)	48.0–63.0	47.0–62.0	45.0–64.0	48.0–60.0	53.0–65.0	52.0–64.0
Minimum, maximum	24–78	28–72	24–75	32–70	29–78	27–76
**Sex, n (%)**						
Male	188 (68.9%)	31 (68.9%)	36 (73.5%)	29 (69.0%)	46 (66.7%)	46 (67.6%)
Female	85 (31.1%)	14 (31.1%)	13 (26.5%)	13 (31.0%)	23 (33.3%)	22 (32.4%)
**Ethnicity**						
Hispanic or Latino	15 (5.5%)	0 (0.0%)	1 (2.0%)	2 (4.8%)	6 (8.7%)	6 (8.8%)
Non-Hispanic or Latino	258 (94.5%)	45 (100.0%)	48 (98.0%)	40 (95.2%)	63 (91.3%)	62 (91.2%)
**Race**						
White	257 (94.1%)	43 (95.6%)	48 (98.0%)	41 (97.6%)	63 (91.3%)	62 (91.2%)
Black or African American	4 (1.5%)	0 (0.0%)	0 (0.0%)	1 (2.4%)	2 (2.9%)	1 (1.5%)
Asian	2 (0.7%)	0 (0.0%)	1 (2.0%)	0 (0.0%)	0 (0.0%)	1 (1.5%)
Native Hawaiian or Other Pacific Islander	0 (0.0%)	0 (0.0%)	0 (0.0%)	0 (0.0%)	0 (0.0%)	0 (0.0%)
American Indian or Alaska Native	3 (1.1%)	1 (2.2%)	0 (0.0%)	0 (0.0%)	1 (1.4%)	1 (1.5%)
Other	1 (0.4%)	1 (2.2%)	0 (0.0%)	0 (0.0%)	0 (0.0%)	0 (0.0%)
Unknown	3 (1.1%)	0 (0.0%)	0 (0.0%)	0 (0.0%)	2 (2.9%)	1 (1.5%)
**Duration of ALS symptoms (months)**						
n	273	45	49	42	69	68
Mean (SD)	19 (8.6)	17 (8.4)	22 (9.4)	17 (8.9)	19 (8.3)	19 (8.0)
Median	18.3	16.5	20.6	18.5	18.6	18.3
(Q1:Q3)	12.0–25.6	11.7–22.7	14.6–28.2	9.9–23.6	12.0–25.2	12.3–24.8
Minimum, maximum	2–40	2–36	7–40	2–36	2–35	6–34
**Site of ALS Onset**						
Bulbar	41 (15.0%)	8 (17.8%)	9 (18.4%)	7 (16.7%)	7 (10.1%)	10 (14.7%)
Limbs	232 (85.0%)	37 (82.2%)	40 (81.6%)	35 (83.3%)	62 (89.9%)	58 (85.3%)
**El Escorial Criteria for ALS**						
Definite	116 (42.5%)	20 (44.4%)	20 (40.8%)	21 (50.0%)	28 (40.6%)	27 (39.7%)
Probable	140 (51.3%)	23 (51.1%)	29 (59.2%)	19 (45.2%)	34 (49.3%)	35 (51.5%)
Possible	17 (6.2%)	2 (4.4%)	0 (0.0%)	2 (4.8%)	7 (10.1%)	6 (8.8%)
**Riluzole History**						
Yes	206 (75.5%)	30 (66.7%)	40 (81.6%)	27 (64.3%)	55 (79.7%)	54 (79.4%)
No	67 (24.5%)	15 (33.3%)	9 (18.4%)	15 (35.7%)	14 (20.3%)	14 (20.6%)
**Concurrent Riluzole Use**						
Yes	189 (69.2%)	32 (71.1%)	38 (77.6%)	29 (69.0%)	45 (65.2%)	45 (66.2%)
No	84 (30.8%)	13 (28.9%)	11 (22.4%)	13 (31.0%)	24 (34.8%)	23 (33.8%)

Abbreviations: mg/kg = milligrams per kilogram; Q1 = quartile 1; Q3 = quartile 3; SD = standard deviation.

## Data Availability

Anonymized data that support the findings of this study are available on request from M.M. (mike@neuvivo.com), representing the study sponsor Neuvivo, Inc. The data are not publicly available due to them containing patient-identifiable information as defined by the Health Insurance Portability and Accountability Act of 1996 (HIPAA) Privacy Rule, the public disclosure of which could compromise the privacy of research participants. Data are unavailable because patient individual data contain patient-identifying details that were subject to Institutional Review Board and Ethics Committee approvals and limitations.
